# Exploring Interprofessional and Self-Compassion Competencies for Applied Behavior Analysis Professionals: A Qualitative Study

**DOI:** 10.1007/s40617-024-00991-5

**Published:** 2024-10-17

**Authors:** Zahava L. Friedman, Daphna El-Roy

**Affiliations:** Kean University, 1000 Morris Avenue, Union, NJ USA

**Keywords:** Interprofessional collaboration, Compassion, Mindful-self compassion, Autism

## Abstract

Interprofessional collaboration, or effective, emotionally responsive teaming between professionals, consists of several skill sets including strong communication skills, compassion and shared knowledge, and can enhance client goal attainment. The field of applied behavior analysis has recently focused on improving capacities of interprofessional collaboration and compassion among its professional workforce. Few studies have reported on perceptions of behavior analytic professionals vis a vis these skill sets in clinical settings. The purpose of this study was to describe participant perceptions of interprofessional collaboration and compassion in the context of applied behavior analytic practice. Following IRB approval, a total of 24 applied behavior analysis practitioner participants were recruited in two cohorts, all of whom participated in a 4-month long training-and-coaching intervention on interprofessional, compassion and self-compassion competencies. Qualitative data consisted of 13 recorded transcripts, including a needs-assessment focus group, as well as each training and coaching session, collected to gain understanding in how participants perceived these competencies. Transcripts were independently coded and analyzed via multistep reflexive thematic analysis by a pair of researchers. Ongoing qualitative analysis yielded the following themes: Historical Perspectives: How We Got Here, More Compassionate to Others Than to Self, Old me versus New me. This study revealed perceptions of barriers and supports embedded systemically in behavior analytic training and culture, affecting development of a collaborative and compassionate behavior analytic workforce. This work highlights the importance of qualitative methodology to enhance research in emerging practice areas through analysis of lived experiences.

## Introduction

Applied behavior analysis (ABA) professionals, such as board certified behavior analysts (BCBAs) and registered behavior technicians (RBTs) work to improve socially significant, observable and measurable aspects of behavior (Behavior Analyst Certification Board [BACB], 2017). ABA professionals serve individuals across the lifespan, with a variety of diagnoses and functional challenges, including autistic, developmentally disabled and neurodivergent populations (Kelly & Tincani, 2013). ABA professionals often work on broad teams of various service providers, including, but not limited to, teachers, speech therapists, occupational therapists, physical therapists, social workers, nurses and psychologists (Friedman et al., 2022). In these cases, interprofessional collaboration (IPC), or individuals from two or more professions working in cohesion, has been linked to improved client outcomes, increased knowledge and efficient service delivery, yet ABA professionals report receiving little-to-no training in IPC (Kelly & Tincani, 2013; LeBlanc et al., 2020; White et al., 2018). Certain studies have highlighted the potential for collaboration between ABA professionals and physical therapists (Fonzo et al., 2020), occupational therapists (Welch & Polatajko, 2016) and speech therapists (Koenig & Gerenser, 2006). Few studies have reported intentional IPC training of ABA professionals via the Interprofessional Education Collaborative (IPEC) recognized training framework, delineating (1) Values/Ethics; (2) Roles/Responsibilities; (3) Communication; and (4) Teamwork/Teams (IPEC, 2023). Other professional groups have historically offered IPEC-based IPC training to students and clinicians, yet until recently IPC within ABA research and practice has had little historical precedence (Taylor et al., 2019).

The core values of ABA may also affect IPC, in that these ethical values guide how ABA professionals perceive collaborative potentiality with other “nonbehavioral” professionals (Brodhead, 2015; Volkers, 2020; Welch & Polatajko, 2016). ABA is built upon observable, measurable behavior change as the gold standard of evaluating effective intervention (Brodhead, 2015). ABA professional consistently report skepticism for nonbehavioral treatments, such as sensory integration treatment and DIR-Floortime, despite working on interprofessional teams where other clinicians actively advocate for and utilize these methods (Friedman et al., 2022; Schreck & Mazur, 2008). The use of the term “nonbehavioral” alongside open assertions in publications and practice regarding interventions from other disciplines as lacking in evidence may hinder interprofessional relations (Friedman et al., 2022; Leaf et al., 2023). Yet, ABA professionals must also adhere to the BACB *Ethics Code* (2020), which delineates evidence-based practice as ethically fundamental. Although certain interventions may be nonpreferred to ABA professional in alignment with the ethical code, it is also necessary to exercise professional humility and interpersonal competence when working with interprofessional partners toward collaboration and client outcomes (Leaf et al., 2023).

The confluence of these factors may be contributing to the perception that ABA professionals struggle with IPC skills; yet, of equal concern is current backlash, both within and outside the ABA profession, of a lack of compassion/compassionate care within ABA practice (Graber & Graber, 2023; Rohrer et al., 2021). Compassion, or loving-kindness in the face of challenges, is an important interpersonal intelligence that includes observable behaviors such as active listening, respectful communication, collaborative decision making and expressing desire to help another (Lown et al., 2015). Compassion is conceptually linked to IPC, as this interpersonal capacity is reported within ABA literature as foundational to teamwork across disciplines, which, from a behavioral perspective, overlaps on several key capacities, such as listening, respect, and shared decisions (Rohrer et al., 2021; Slim & Reuter-Yuill, 2021; Taylor et al., 2019). Compassion is foundational to understanding how others, including families perceive and experience ABA interventions and ABA professionals as interprofessional and clinical partners (Brown et al., 2022; Graber & Graber, 2023). As Rohrer et al. (2021) pointed out, though collaborative competencies can exist without compassion, compassion *in service of* collaboration is a more effective approach to team-building across individuals.

Compassion in clinical settings can be operationalized into specific skills that are teachable, such as mindful attention to individuals served, active and focused listening and direct action to support the individual toward their goals (Lown et al., 2015). Although compassion can be a compatible competency supporting IPC, it can also be turned toward oneself, to face one’s own suffering in a compassionate way (Neff et al., 2021). Self-compassion, or compassion toward oneself, is not only a capacity; it is a skill that can be operationalized, trained, and practiced for improvement (Neff et al., 2021). In the Mindful Self-Compassion (MSC) framework, three principles of teaching and learning self-compassion are embedded: (1) Loving, or loving oneself; (2) Connected, or understanding that mistakes are part of the human experience; and (3) Presence, or mindful, gentle awareness of one’s emotions (Germer & Neff, 2019). An emerging body of knowledge supports use of MSC framework in building self-compassion and collaboration across organizations (Neff et al., 2020; Tabancali & Öngel, 2020). As Neff et al. (2020) point out, improving self-compassion can complement, rather than detract from, compassionate care, because increased self-compassion is associated with reduced stress and improved overall well-being. Clinicians who operate from a space of wellness may also be more available to dedicatedly and compassionately support the individuals they serve.

Despite the potentiality of targeted, emotionally informed trainings, educational offerings for ABA professionals that are collaborative and *also* compassion/self-compassion focused have not been well-documented in the research (Lown et al., 2015; Tabancali & Öngel, 2022). Although attempting a practice shift toward a more compassionate ABA approach, it is important to also include viewpoints of ABA professionals, because they work to build both IPC, compassion and MSC competencies (Neff et al., 2021). The current study, therefore, describes lived experiences of 24 ABA professionals, as they engaged in a 4-month-long training and coaching intervention in IPC compassion and self-compassion. The purpose of this study was to synthesize ABA professional’s descriptive data pertaining to perceptions of their personal capacities of compassion and collaboration within ABA contexts. As Burney et al. (2023) stated, qualitative methods can complement the traditionally quantitative ABA research base, to achieve improved social validation while expanding research into emerging practice areas.

## Method

### Participants

Upon IRB approval from a northeastern U.S. university IRB, a total of 24 participants (*n* = 24) were recruited, all of whom were verified as meeting delineated inclusion criteria of “registered behavior technician,” “board certified assistant behavior analyst,” or “board certified behavior analyst,” currently employed in that capacity. Participants were recruited through two employers, where training and coaching were provided virtually through the workplace as part of scheduled professional development during the work day. Participating agencies were recruited using the first author’s professional network, in accordance with IRB-approved methods. Two employers agreed to serve as community partners in the delivery of the training-and-coaching sequence: one on the East Coast of the United States and one in the Pacific coastal U.S. region. Both employers owned agencies where children, adolescents, and adults with a documented autism/autism spectrum diagnosis received a variety of center-, home-, and school-based services through those agencies. Eleven participants (Cohort A) hailed from the east coast, ranging in age from 22 through 52, with an average age of 36.8 years. Thirteen participants (Cohort B) hailed from the West Coast region, ranging in age from 19 through 45, with an average age of 28.53.

All participants from the East Coast identified as female, whereas 4 of the 13 from the Pacific coastal cohort identified as male and 9 of the 13 as female. Recruitment commenced upon IRB approval and was planned to continue until a minimum of 15 participants were recruited, in accordance with researcher hypothesized sample size necessary for qualitative data saturation. Following participation from both sites, a total of 24 participants were recruited. Though an additional wave of recruitment was considered in January 2023, researchers elected to first analyze available data, which subsequently saturated around the themes outlined later in this article. Demographic characteristics are included in Table 1.

**Table 1:** Participant Demographics

Study Labels for RBTs	Age, Sex	Primary Area of Practice	Study Labels for BCBAs	Age, Sex	Primary Area of Practice
1 RBT 1	22, female	East Coast, clinic-based	1 BCBA 1	41, female	East Coast, clinic-based
2 RBT 2	24, female	West Coast, clinic- and school-based	2 BCBA 2	29, female	East Coast, clinic-, school-, and home-based
3 RBT 3	26, male	West Coast, clinic- and school-based	3 BCBA 3	52, female	East Coast, clinic-based
4 RBT 4	27, female	West Coast, clinic- and school-based	4 BCBA 4	30, female	East Coast, school- and home-based
5 RBT 5	22, female	West Coast, clinic- and school-based	5 BCBA 5	47, female	East Coast, clinic- and school-based
6 RBT 6	29, male	West Coast, clinic- and school-based	6 BCBA 6	28, female	East Coast, clinic-based
7 RBT 7	19, female	West Coast, clinic- and school-based	7 BCBA 7	43, female	East Coast, clinic- and school-based
8 RBT 8	28, male	West Coast, clinic- and school-based	8 BCBA 8	45, female	East Coast, clinic- and school-based
9 RBT 9	27, female	West Coast, clinic- and school-based	9 BCBA 9	31, female	East Coast, clinic-based
10 RBT 10	35, female	West Coast, clinic- and school-based	10 BCBA 10	37, female	East Coast, clinic- and school-based
11 RBT 11	30, male	West Coast, clinic- and school-based	11 BCBA 11	45, female	West Coast, clinic- and school-based
			12 BCBA 12	24, female	West Coast, clinic- and school-based
			13 BCBA 13	35, female	West Coast, clinic- and school-based

### Intervention Procedure

Data were collected from August 2022 through January 2023. A primary investigator (PI) and co-investigator (co-PI) co-led all aspects of the study, including recruitment, training, coaching, recording, design, and analysis, to support consistency across delivery of the intervention. As far as positionality, the first author (PI) was dually certified and licensed as an occupational therapist and BCBA, with a working background in pediatric home-, clinic-, and school-based settings, and further had previous publication and research experience in interprofessional/IPEC rooted scholarship. The second author was certified as a BCBA-D, identified as an ABA academician, and had previous experience in schools, ABA clinics, home-based settings, and on interdisciplinary teams, as well as with peer-reviewed ABA intervention research. The study was delivered in a cohort model, with two cohorts (Cohort A and Cohort B) completing the training-and-coaching sequence in tandem. Participant data were only available to the two investigators throughout and subsequent to the study. Participation was voluntary; training and coaching sessions were provided as free professional development opportunities to all employees of a pair of ABA companies. Employees who chose not to participate from within those companies did not attend training or coaching sessions or chose to attend but remained off camera and silent.

As participants were recruited from these two ABA agencies based on availability and feasibility, convenience sampling on a rolling and snowball basis was utilized through agency communications. This pattern of recruitment was also selected based on the increased investment required to design and implement the intervention sequence. Recruitment was paused after the completion of two cohorts to allow for the researchers to properly analyze results and thereby evaluate program effectiveness, while assessing whether qualitative data could reach saturation. Delivery of all interventions were provided to participants as a cohort, with equal access and content available for both RBTs and BCBAs. This was designed intentionally to model a spirit of information sharing, collaboration, and compassion within the agency in alignment with the content and values embedded in their program. Also, although RBTs and BCBAs are two clinically distinct groups, the demands placed on them from an ethical and soft-skill standpoint are complex, necessitating training for both RBTs and BCBAs. In certain cases, the RBTs who agreed to participate worked with or under BCBAs who also agreed to participate, though participation was not mandatory for BCBAs or RBTs, and some employees from each workplace chose not to participate in the study.

All participants completed informed consent to participate in the study as well as informed consent for audiovisual recording of the training and coaching sessions. Some employees chose not to participate, and were therefore, not included in the study or any collected recordings/data. All training and coaching sessions took place on Zoom video-conferencing platform, with a secure, distinct link shared prior to each session, and a waiting room in place to screen employees who had completed informed consent and agreed to participate. Only individuals who had signed informed consent and who were recognized as participants were admitted into the virtual space by the PI and co-PI. Following informed consent completion, professionals from Cohort A were invited to participate in an initial, optional focus group to assess need and areas of priority for the training session; Eleven participants attended that needs assessment. At this point, the training was designed, informed by qualitative analysis of focus group data, which revealed prioritization of interprofessional collaboration and self-compassion techniques in terms of utilizing practical strategies and problem-based learning. All subsequent aspects of the study were guided by this needs assessment, while continuing to align with ongoing feedback from participants.

Because the rolling and snowball recruitment pattern was utilized, Cohort B was recruited following the initially recorded needs-assessment focus group, with training and coaching sessions for each cohort occurring independently. In the case of Cohort B, a general, nonrecorded needs assessment occurred at the organizational level prior to participant recruitment, with clinical leadership from that organization describing the needs of that cohort. Needs assessment data underwent an initial analysis by both researchers, subsequently leading to design of the training content to align with participants’ needs and priorities. Coaching was conducted individually for Cohort A, and in a group format for Cohort B; differences between cohorts were designed as a collaboration between researchers and agency administrators based on need, availability of professionals, and preferences of the participants from each agency.

Coaching sessions included a general script, with additional open-ended responses encouraged from participants, through questions such as, “What are some challenges in collaboration that you’ve experienced in the past week or month? How would you describe your handling of these challenges? How did you feel about these experiences?” Some participants reflected on clinical examples as well as attempts and use of previously reviewed self-compassion and interprofessional collaboration strategies. Participants were also given the opportunity to ask questions and request review of certain skills/practices. Due to potentially sensitive content of the study, it is important to note that specific preferences were also honored to ensure that participants felt comfortable in co-designing and collaborating with researchers in the process of intervention delivery.

Following ICF completion, all participants then participated in the needs assessment (Cohort A) or in the initial training session (Cohort B). During the needs assessment (and subsequent coaching opportunities) a semi-structured script was followed, based on review of the ABA literature on compassion and collaboration, and more specifically delineated by Friedman et al. (2024). This semi-structured script followed grand-tour and probing questions of the target topics, including interprofessional collaboration experiences, perception of compassion competencies in ABA practice and orientation to self-compassion work (Germer & Neff, 2019). The training session consisted of an interactive workshop covering interprofessional collaboration and self-compassion models alongside experiential learning activities. Active learning opportunities embedded in this training included reflections, discussions, role plays, and applicable case study review by peers. Rather than utilizing a strict lecture format for the training, researchers offered multiple avenues for participants to engage actively with content, through reflective questioning, encouraging articulation of concerns, and allowing for written (chat), verbal, and poll responses. In this way, each participant was encouraged toward active learning during the training, by responding to each other/the researchers, engaging in role-play exercises, reflecting on probing questions and through experiential breathing practice. Although all participants responded both verbally and in the chat, certain participants elected to share or participate more than others, based on personal level of comfort.

Coaching sessions consisted of participants sharing personal and/or professional challenges, with researchers following a similar coaching structure in each session. Following completion of the training and two coaching sessions, participants completed poststudy reflections via focus group and/or survey, depending on self-selected method of reflection (both were offered). When both cohorts completed the full training and coaching sequence, recording transcripts were cleaned and analyzed, with independent coding by the PI and Co-PI, followed by consolidation of codes and then a proposed thematic meaning. Please see Table 2 detailing the study timeline, noting differences in timing/delivery between the two cohorts.

**Table 2:** Study Timeline and Activities: Training and Coaching Sequence

Study Phases	Timing of Cohort A	Timing of Cohort B	Outline of Activity
Phase 1: Focus Group, Needs Assessment (1–2 h)	August 2022	October 2022	Meeting to assess areas of need in terms of collaboration, compassion and self-compassion. Operationalize collaboration, compassion and self-compassion into discrete skill sets to inform future training
Phase 2: Training (3–5 h)	September 2022	November 2022	Seminar style training on skill sets of collaboration, compassion and self-compassion
Phase 3: First Coaching Session (1–1.5 h)	October 2022	November–December, 2022	Review and coaching with follow-up questions, re: individual sharing of challenges/successes in collaboration, compassion and self-compassion
Phase 4: Second Coaching Session (1–1.5 h)	November 2022	December 2022	Review and coaching with follow-up questions re: individual sharing of challenges/successes in collaboration, compassion and self-compassion; reflections on growth and team building
Phase 4a: Posttest/ Reflection (1–2 h)	December 2022	December 2022–January 2023	Open-ended survey and open-ended focus group questions pertaining to content and delivery of training and coaching intervention

Detailed training, coaching and needs assessment content have been previously published (Friedman et al., 2024)

Because this article’s purpose was to describe perceptions, readers are encouraged to utilize the aforementioned study to review detailed intervention methodology

### Data and Data Analysis

Data analysis commenced following completion of intervention for both cohorts, over a 6-month time period from January through June 2023. For this study, the qualitative tradition was intentionally selected, to gather perspectives and lived experiences in a way that quantitative analysis or single-study case study may not be as well-suited (Burney et al., 2023). Qualitative data consisted of all recorded transcripts (13 transcripts in total, ranging from 30 to 180 min in length), including needs assessment, training, coaching sessions and poststudy focus groups. Though Zoom transcription produced an automated transcript as an output of each virtual meeting, the PI and co-PI reviewed all transcripts for accuracy, editing to match verbiage to the audio-visual recordings of each meeting. The initial group needs assessment (Cohort A), and all training and coaching sessions (Cohorts A and B) were recorded, transcribed, and reviewed/cleaned for accuracy. Video recordings and audio transcriptions were independently coded and analyzed by the team of two researchers for verbal output (transcript) data, observable physical reactions (video), and tone of voice (video). Reflexive thematic analysis (RTA), a qualitative methodology that honors the reflexive and embedded role of the researcher within the analytic process, guided the analytic process (Braun & Clarke, 2019; Campbell et al., 2021). RTA, delineated as used within this study in Table 3, involves a several-step process of delineating commonly occurring words/codes, condensing these into ideas/themes, and synthesizing the data into the larger story (Campbell et al., 2021).

**Table 3:** Delineation of Reflexive Thematic Analysis, per Campbell et al. (2021)

Analytic phase	Description	Action
Data familiarization	In-depth data immersion, begin looking for patterns	Transcription, correction Multiple readings of the transcription with note taking/ recording of analytic memos
Initial Code Generation	Considering the full transcription as a whole, creating plentiful and rich codes to make meaning of phrases	Labeling phrases/sentences by groups and categories
Generating (initial) Themes	Combining or elevating codes to gather into initial themes Naming themes and defining their individual and inter-related meanings	Thematic diagrams/maps Writing and describing themes
Theme Review	Reviewing patterns of codes by re-reading transcripts Reviewing entire data set as a whole	Ensuring there are plentiful quotes and data to support each theme Remove/collapse overlapping themes Refining and drawing greater insight from existing themes
Theme refining and Naming	Delineating the greater story and the place of each theme in it Connecting the findings to the research question or aim	Reviewing data, codes and themes as a whole, as well as original research aim to assess connectedness

The PI and co-PI each independently coded all transcripts via open coding. As stated, each author had previous experience as certified BCBAs (one was also certified as an occupational therapist) working on interprofessional teams, attuning researchers to the unique language underpinning the experiences of interprofessional practice. Because in RTA the reflexive thought processes of the researcher are honored, it is crucial to understand the background of these researchers as having previous experiences working on interprofessional teams, driving this work and its analysis forward. This was followed by researchers meeting for several months to engage in debate regarding codes and analysis of the data, to emerge with axial or combined codes that reflected further analysis and synthesis. Researchers met and continued to consolidate codes and analyze transcripts until 100% researcher agreement was reached regarding codes and themes (Braun & Clarke, 2019). Though agreement among coders was not strictly aligned with RTA, it was deemed necessary due to the sensitive and complex natures of these topics. Qualitative themes describe the reflective experiences of the 24 participants with regard to the intervention, as well as their perceptions of collaboration, compassion and self-compassion as practice-supportive constructs.

## Results

Twenty-four ABA professionals participated in this study. Based on ongoing qualitative review resulting in thematic saturation within this data set, three general themes emerged from the data as follows: (1) Historical Perspectives; (2) More Compassionate to Others Than to Self; and (3) Old Me versus New Me. These themes demonstrated shifting participant perceptions, as engaging in collaboration and self-compassion practices revealed prior challenges (Historical Perspectives theme), current learning (More Compassionate to Others Than to Self), and programmatic-related growth (Old Me vs. New Me). As noted in Table 3, thematic analysis emerged from consolidation of pertinent codes, while conceptually connecting to training-and-coaching elements intentionally embedded throughout the study. Though initially revealed as four themes, the researchers subsequently conceptualized findings into a three-theme format, as two themes held similar meaning and were therefore combined into one. In this table a “lens” refers to an understanding of participant quotes through the viewpoint or filter of various training concepts, serving to delineate how these training concepts were perceived and internalized by participants, supporting personal growth. Each theme, therefore, along with relevant quotes and explanations, is delineated further in Table 4 below, with subsequent explanations.

**Table 4:** Conceptualization of Codes and Themes

Theme	Contributing Codes	Self-Compassion Lens	Collaborative Lens
Historical Perspectives: How We Got Here	-The BCBA who raised me -Calling Training the “Upbringing” -Personal History stories of challenge -Using my personal experiences as a mom, child etc -independence from a young age -I used to be dismissive -OT/SLP has no real role -little/no training in collaboration	**Loving** Nonjudgmental, compassionate self-reflection and awareness of how each individual’s history and training contributed to challenges in collaboration	**Communication/Collaboration** Movement from previous collaboration-avoidant state to collaboration-accepting/embracing state via self-awareness. Acknowledging pieces in my past that were barriers to collaboration
More Compassionate to Others Than to Self	-I’m always the one reaching out -Type A -So hard on myself -I need to let it go -Kicking myself for mistakes -Like-minded women -I’m kind to everyone but myself -No one even asks me -more compassionate to others than to self	**Connected** Learning to accept challenges, understanding that everyone can make a mistake including us as practitioners and as individuals	**Role Perception** Understanding clearly what the role of the behavior analysis practitioner is, while also delineating and learning about the roles of others on the team. I don’t need all the pieces so I can be easier on myself
Old me vs. New me	-Collaboration as the effective solution -We can’t make progress without the team -We need the family on board -Teachers and OTs schooled me -Don’t blame, just make the change -saying the same things, different words -Clear is kind -now I notice when I have challenges	**Presence** Noticing and naming previous and current challenges to become self-compassionate during those challenges	**Conflict Resolution:** Observing current collaboration conflicts, from both internal reaction and external events (perspectives of others) viewpoints

## Theme 1: Historical Perspectives: How We Got Here

ABA professionals have historically been educated, trained, and supervised in multiple content areas, but the value of and ways to collaborate with other professionals have not been traditionally emphasized, if addressed at all. Although there is nothing inherently negative about professional pride, among the behavior analytic workforce ABA methods and frames had been considered an all-encompassing, ABA-answers-all approach. Per participant perspective, this viewpoint regarding supremacy of ABA inherently created a collaborative barrier. BCBA 6, BCBA 1 and BCBA 7 shared their histories. BCBA 6 said, “Behavior analysis is the... way, the truth and the.. light, and so most of my... grad school education was... zero focus on collaboration.” BCBA 1 shared, “I've been doing this for a long time... and I would say that.. I feel like... a lot of us had the same training... when you were an instructor, it was very much like this is what we're doing... like *our way or the highway,* you know?” BCBA 7’s experience was,

The behavior analytic training [was] *behavior analysts know it all*. I remember somebody once asking me. Well, if you're teaching this, then why do we need a speech therapist? And then my answer is sort of being like, well, you don't. You know so not . . . the way to be at all. And I think it was my experience with that one class and that one professor. That was basically [it]. We all have an enormous amount of education and training, and you can't be so dismissive of that, and I think . . . that it took another nonbehavior analyst to kind of bring me to that.

This acknowledgement of professional background and critical reflection on her own dismissive reaction demonstrates the shift in her perspective on collaboration. Furthermore, reflective understanding of “nonbehavior analyst” language was revealing, as both individuals and approaches that were “nonbehavior analytic” were repeatedly verbalized by participants. This underscored the historical context of members of corresponding professions being seen as “other” or “non.”

The lack of structured, formal training regarding other professionals extended to working with families as well, and the insight to the importance of that collaboration came from personal experience. BCBA 1 said,

I didn't have any . . . training about how to really be compassionate with families. I think a lot of my learning came from having my own children and then saying like oh, my goodness, this is really hard and then trying to do a better job of preparing people that I worked with to say like it is actually not realistic.

This shows that despite the lack of professional training in this regard, personal experiences changed participants’ viewpoints in their interactions with families. ABA professionals talked about previously discouraging families from nonbehavior-analytic interventions, and later initiating a conversation with families to determine flexibility to maximize effectiveness of treatment. BCBA 1 also shared,

I would just say, you know, when I first started in this field so many, many years ago, I feel like we had . . . you know those types of things where what we saw modeled for us was you know basically saying to families there’s no evidence there’s no evidence there’s no evidence.

Although criticism of other disciplines’ interventions impeded collaboration, participants also shared that the expectation to use precise ABA-professional terminology within the profession actually impeded communication with families/professionals unfamiliar with this jargon. Participants described a need to “code-switch” between the language of behavior analysis and describing familiar procedures to clients or professionals from other disciplines. Participants shared personal struggles with creating a shared language with professional partners and families served, with the high expectations of an ABA professional acting as a barrier of sorts to communication outside the profession. BCBA 7 said,

My training was . . . you have to speak precisely and if you're not saying controlling variables of the mand . . . MO specific reinforcement . . . topography-based versus selection-based verbal behavior . . . you're using all these words . . . if you're not using that then you're not good enough.

Not being considered good enough within the profession if terminology is not used and communicating well with professionals from other disciplines was revealed as a personal barrier to collaboration. BCBA 9 said, *“*We're not always talking about the same exact thing, but we're in a lot of schools and a lot of times people are talking about similar things or trying to get it similar things, but using different terminology.”

An interrelated concept was revealed to be a strong valuation of independence, as ABA professionals trained to believe in the supremacy of ABA principles described a feeling of “needing to do it all.” Although professional and personal independence was valued among participants, they also shared that when these values were taken to the extreme they may have caused undue pressure. In this vein, BCBA 3 revealed a dawning awareness of the importance of asking for help, stating, “I've learned it's easier to do it yourself... than when it is coming to asking for somebody else for help.... I think I have learned asking for help is not a sign of weakness it makes me be it actually frees me up to do more.”

## Theme 2: More Compassionate to Others than to Self

Analysis of transcripts and open-ended survey responses revealed the consistency with which participants described their compassion toward others being greater than their self-compassion. In the poststudy survey, one participant’s response included, “I tend to think I am compassionate in most situations towards others. Self-compassion is not something I had ever thought about and it has become an important piece of my daily work.” Another participant stated in the survey, “We all feel inadequate and are hard on ourselves. I can have more compassion for myself now.” Professions are said to attract certain people, and ABA is no exception. Qualities of individuals attracted to the ABA profession may be professional strengths, yet these qualities can also have undesirable presentations or aspects. BCBA 3 succinctly shared, “We are all like-minded. We're all the same. We're driven, you're gonna get it done.” BCBA 7 said,

We think alike and we talk alike. Well, I think that we all attract similar types. Very type A. Very goal oriented, very determined individuals and like we have patience of saints for anybody but . . . myself like I am ridiculously hard on myself.

BCBA 4 likewise shared, “I think a lot of us here we’re very like type A. We’re impatient . . . we're goal oriented.”

These quotes highlight a strong sense of responsibility held by participants, while describing how these leadership qualities could have led to undesirable results. BCBA 4 also shared, “*Holding the responsibility of being the bridge of collaboration*, it’s a big responsibility... no one else is reaching out... I’ve always been the one to initiate it.”

Regarding facing challenges to collaborate, BCBA 1 discussed,

Siloing . . . happens . . . people are very rigid about what their role is…and not in a way where I don’t want to step on your toes more in . . . a way where we’re going to blame-shift if something doesn’t go well . . . one of the things I try to communicate to families is one of our jobs . . . is to be a bridge between all the different providers which is a big responsibility to take on.

BCBA 2 noted,

Last week I had someone reach out to me. It was honestly the best conversation I’ve had with a provider. . . . I wondered too if it was partly because they reached out to me. In the past I’ve always been the one reaching out to other providers, and those conversations don’t always go in the best direction. . . .

Being part of a team changes the expectation of carrying the burden alone. BCBA 4 revealed emerging self-awareness, saying, "I don't have the answer but perhaps I can be a piece that helps to get this child a better life.” BCBA 13 talked about the whole being better than the parts, a learning concept presented in the training and reinforced throughout, when she said, “I like the idea of not trying to be an expert in... collaborating and staying a learner. I think that just stays useful to the team. because then it kind of keeps everybody in like a cooperative, collaborative mindset.” Multiple participants talked about giving up control and perseveration on mistakes. BCBA 4 said, “normally would have sat with me... why can’t I get that right?” BCBA 7 also shared, “[I] had to let things go and accept what was important.”

ABA professionals talked about themselves as more compassionate to others than to self, both personally and professionally, as they learned about self-compassion strategies and worked on applying them. Self-compassion exercises introduced during the training provided in this study involved talking to yourself as if you were talking to a loved one and writing a letter to yourself as if it was a letter to a loved one, which unearthed the complexities of relationships with oneself and others. RBT 2’s letter included:

Dear RBT 2, take some breaths and apologize for accidentally yelling at your boyfriend or your mom, and share that it's because you're frustrated with how your day at work went, admit your fault that you're sorry for your freaking out and move on. Everyone has bad days, but don't take it out on the people you love when they just want to hear what you went through, or hear what you have to say. You have long days, and are always busy pleasing everyone before yourself that you don't give yourself the time to debrief your days. . . .

BCBA 9 admitted,

I certainly . . . try to be self-compassionate. . . . I think that that's what stuck with me that you were like talk the way you would talk to, you know, like a good friend or a loved one, like you wouldn't you wouldn't tear them down so, you know, why do it to yourself, that's kind of where I've been trying to, trying [to] be. Trying. Is the keyword.

RBT 3 said,

I think there was a part where, like you like when you were trying to get that self-compassion, and the most easiest one is to think about someone that you love, or someone that you care and think about all the things that you want to say to them, make sure that you apply that to yourself.

ABA professionals’ training focuses on analyzing the environment to make changes to benefit individuals, rather than blaming individuals. But this training does not necessarily generalize when ABA professionals respond to their own mistakes. BCBA 7 said, “I think I accept responsibility for like all things gone wrong.” BCBA 4 said,

You know how many times I have said this to my RBTs, to teachers, to anybody I'm working with like you can't blame the kid’s behavior on the kid. You have to blame it on the environment. What's going on in the environment? Let's change it to fix the problem. But never once have I thought of it that way for myself and never once have I really even thought about those questions before taking that survey. To be quite honest, I graded myself pretty harshly, but I have definitely been in situations where something didn't go the way I wanted it to, or something bad happened and I will harp on it for days like it'll ruin a weekend. I can't stop thinking about it, I'm dreaming about it, and ultimately I do get myself to kind of calm down and come to a solution, but not without first, like we all say, we have the perseveration trait, without like perseverating on it for a long period of time before you kind of get to that acceptance.

In the poststudy survey, BCBA 8 responded, “Self-compassion is a critical feature that I need to work on in order to be a more effective clinician and more importantly, more tolerant of my mistakes. The resources that have been shared during this process makes me feel empowered that I can continue to learn and grow,” When asked what they learned and in response to what resonated with them that they felt they could consistently implement, RBT 9 wrote, “Speaking to myself, the way, we speak to others when they make mistakes and utilizing some physical touch to oneself to help relax and calm down.” RBT 4 noted, “... everyone fails sometimes.”

## Theme 3: Old Me versus New Me

Participants shared scenarios they would have handled differently in the past and the rationale and how noticed a personal shift in their response toward a more collaborative, compassionate, and/or self-compassionate outlook. BCBA 2 and BCBA 4 shared changes in their interactions with parents. BCBA 2 talked about currently supporting a mother’s consideration of discontinuing services, rather than trying to convince her to stay, as she would have in the past. She said, “I felt really good because I feel like 3 years ago, when I started as a BCBA, I would have tried to convince this mom to stay.” BCBA 4 mentioned that she liked this quote from the training, “I’m never going to accomplish my goal without you. And I feel like I'm gonna start saying that to parents.” ABA professionals recognized the importance of collaborating with the parents to be successful, also, how this may not have been evident to them at the start of their professional careers.

Communication was a central theme of discussion and shift. BCBA 1 tied clearer communication to kindness when she said, “If I’m being unclear then I can't hold anybody accountable because I wasn’t clear. So now I’ve been clear, now I’ve been kind, now, you know, these are my expectations, and you can meet them. But if I haven’t been clear, then I can't hold anybody you accountable for not meeting my expectation.” BCBA 6 said, “... trying to understand, OK, what perspective are they taking? Where are they coming from and just trying to like dive into it a little bit deeper to ask questions about it rather than being dismissive of it.”

After working in professional settings, there was also a shift in the approach to interacting with colleagues from other disciplines to a more collaborative one. BCBA 3 said, “I was schooled... by this brilliant OT and wonderful speech pathologist who said, ‘I’m not going to be your TA.’ I have something important to say and... you know, and it... did turn out amazing where I felt like I learned a lot about other professions....” In the poststudy survey one participant reflected about learning, “Respect other professions and always be open to hearing another perspective that can be helpful to the team....” In the poststudy survey one of the participants replied regarding strategies that resonated with them and they felt they could consistently implement, “Recognizing I am a piece of a puzzle, be curious not judgmental of other professions.”

One of the strategies discussed in the training involved using the phrase “I notice/d.” This specific strategy was shared from the self-compassion skillset, and appeared to support both compassion and collaboration. BCBA 11 discussed this regarding her own family interactions. She shared an interaction with her family,

I think for me, one thing that I learned is like I've been using that phrase “I notice,” I've been testing that one a little bit so that was a helpful one. And what I found really interesting in my family because . . . got the last word in, you know. Gotta like, hey? if I rib you, That means I love you kind of ways of communicating, but like sometimes it's not really welcome. And I notice when I say ha I notice that's a little meta, I notice when I say that, then it isn't like the same there isn't the same need to have the last word in by the receivers of that, sentence it takes some of the power struggle, *that’s not even the right word, *but it takes some of that out. *It takes some of that out, it's just focused on like wait, something about what's happening isn't working for all parties here.*

The responses to the training provided were positive, and multiple participants identified a collaboration and or self-compassion strategy they practice and utilized in personal and clinical settings. They also shared that although they may not have practiced or valued these soft-skill competencies at the onset of their professional careers, over time they grew to appreciate and understand their importance.

## Discussion

This study highlights the unique and complex orientation of 24 ABA professionals to soft-skill competencies in the context of ABA clinical practice. Participants described their experiences with regard to how collaboration, as a construct, had historically and personally been perceived, by themselves and others within the ABA profession. In the first theme, the specific “upbringing” of ABA professionals, per participants, had included some form of avoidance or dismissiveness of IPC, relating back to research pertaining to lack of training of ABA professionals in collaborative competencies (Friedman et al., 2022; Kelly & Tincani, 2013; LeBlanc et al., 2020). Throughout the history of the profession ABA professionals have also shared and lamented lack of training in cultural diversity/humility (Beaulieu et al., 2019) and compassion (Taylor et al., 2019), whereas recent literature has highlighted the need to address these skills in practice settings (Rohrer et al., 2021; Slim & Reuter-Yuill, 2021). This research demonstrates the importance of centering the conversation about historical precedence within ABA as a field in order to support individuals and groups of practitioners toward a more self-compassionate approach toward the self. Though compassionate care and IPC may present challenges for each person, understanding the historical culture as a contributing source of personal struggles (vs. isolated within a practitioner) can enable a gentler orientation to oneself. It is important to note that although BCBAs in this study tended to have a stronger insight into the systemic mechanisms behind the lack of emphasis on compassion within their training histories, RBTs acquiesced by verbally agreeing with statements made by the behavior analysts.

The second theme pertained to a perception among participating ABA professionals that each individual demonstrated greater compassion to others, such as clients and coworkers, rather than to themselves. There was a uniformity with how hard-driving ABA participants were on themselves; they had a strong sense of responsibility to individuals served, often committing to solving problems on their own (even outside their scope of practice). This became problematic with particularly hard cases, where setbacks and conflicts arose with regard to role perception (with ABA professionals and other interprofessional partners overstepping role boundaries) and acknowledging errors or mistakes. The ABA literature discusses ethical challenges of nonbehavioral or non-evidence-based treatment, language that although accurately describing methods as outside behavior analytic principles also judges these approaches conceptually as *other* or *less than* (Brodhead, 2015). Participants used similar language throughout the study, as they described navigating the ethics of IPC, when approaches from other professional fields such as occupational and speech therapy were openly challenged in clinical settings. In an ideal situation, role perception can *improve* IPC when representatives from various disciplines create and contribute to shared goals in collective knowledge contributions (Slim & Reuter-Yuill, 2021). Because collaboration is currently a vital competency in the BACB *Ethics Code* (2020), and role delineation is interrelated to that competency, handling the boundary-filled role landscape of a team remains vital to one’s professional functioning. The data analysis revealed that both BCBAs and RBTs felt an intense personal drive and responsibility toward excellence, underscored by how and what they shared during the training and coaching sessions.

The third theme perhaps most strongly demonstrated the potentiality of the training-and-coaching sequence to affect mindset and culture shifting, as ABA participants compared their former orientation to IPC and self-compassion to their developing current and future selves. By the last coaching session, participants were able to engage in a more vulnerable and honest manner, sharing openly their emotional states, feelings, and thoughts. The ABA profession centers observable and measurable behavioral outputs; therefore, within this context it is important to note that the skills of IPC, compassion and self-compassion can be observed and measured, in terms of frequency, length, and content of emotional awareness (Slim & Reuter-Yuill, 2021). The emerging behavior-analytic literature has established a need for expansion of soft skills (Rohrer et al., 2021, Taylor et al., 2019), including collaborative and compassionate competencies. As Leaf et al. (2023) has asserted, working on interprofessional teams requires expanded and more flexible clinical and interprofessional approaches to prevent conflict in these settings. The current study adds insight as far as what specific improvements in collaboration and self-compassion can be operationalized as observable and measurable outputs of soft-skill expansion training. The researchers in this study observed a particular shift, led by the BCBAs and followed subsequently by RBTs, in the behavior of participants openly sharing challenges; just as BCBA’s provide clinical leadership in behavior-analytic service delivery, their leadership in soft-skill practice within this study was evident.

## Implications for Research and Practice

This study presented several implications to guide future research. First, future research can embed other professional clinicians, such as speech, physical, and occupational therapists, to continue to build collaborative relationships between groups of professionals. It may also be helpful to include administrators or leadership from ABA clinics or organizations in order to promote interprofessional and compassionate practices at all levels of an organization. Future focus groups can include professionals from other certified areas in order to continue to build understanding of perceived strengths and barriers in how the ABA profession is perceived, as well as areas for growth. Research questions that were not addressed in this study can guide further inquiry pertaining to building compassionate structures with family members through active training and coaching that intentionally includes the family. In addition, building and implementing an operationalized set of observable behaviors pertaining to collaborative and self-compassionate competencies through the behavior analytic lens is an important future target. It may also be important to separate RBT and BCBA participant groups in future training, though the current study demonstrates the advantages of providing RBT and BCBA mixed cohort training, including time effectiveness, intra-professional conversation, and workplace-wide consistency.

As far as practice settings, participants in the current study describe an emerging orientation to their clinical practice that benefitted from engaging in MSC exercises and coaching in collaborative problem solving. This demonstrates the importance of ABA professionals pursuing a study of improving self-awareness, as a component of building IPC with other professional groups. Current practitioners may also benefit from actively engaging in interprofessional education opportunities, through case and knowledge sharing in their workplaces and in community with colleagues. Because this study utilized agency-supported recruitment, this highlighted the benefits of bringing IPC training at the organizational level, through engagement of leadership at the early stages of implementation. The IPEC competencies have been well-established across medical and therapeutic literature and can be more intentionally embedded in ABA college and workplace training (IPEC, 2023). Bringing supportive leadership into the conversation also revealed an inherent support to collaboration within an organization: when there is a cultural *openness to and belief in* IPC, this mechanism is a strong driver of change. Finally, this study revealed the inherent individual perception in how improvement occurs within clinical soft-skill competencies; therefore, each individual practitioner can (and perhaps, should) engage in and with these topics with a personal sense of responsibility toward their own unique growth.

## Limitations

Limitations refer to unavoidable aspects of a study that limit generalizability of its results. The current study recruited participants from within two organizations; support from leadership may have biased participants, predisposing them to engage due to existing power structures within their workplaces. Further, BCBAs and RBTs both participated in this study as mixed cohorts, despite their varied roles, expectations, and training experiences. It is possible that existing workplace or credential-based power dynamics existed within the study between BCBAs and RBTs who worked with them, biasing the study results. Also, this training did not differentiate between RBTs and BCBAs in content or delivery, potentially limiting its application to both groups while not customizing for either. The participant pool was intentionally small. Although this was designed in order to build rapport, remain feasible to complete, and maintain fidelity to intervention delivery, it is possible that results might have differed with a larger group of participants.

Further, all intervention was delivered virtually, potentially creating inequities during coaching sessions for participants who may have experienced technology limitations. No face-to-face contact occurred, limiting generalizability of perceptions to similar training provided in person. This study lacked follow-up after the training-and-coaching sequence was completed. Therefore, it was not determined how participants perceive the intervention in the long term. Results consisted of qualitative data, which at the methodological level may contain its own bias due to subjectivity and inherent pressures within the study. Finally, no work products were collected, making it difficult to determine what measurable and behavioral effects resulted. Although this data collection method was aligned with the study’s purpose, it did not include any direct measure of behavior change.

## Conclusion

This study revealed the perceptions of participants regarding collaborative and compassion competencies in ABA practice. Through qualitative analysis of responses, systemic barriers, and supports to building a compassionate, collaborative ABA workforce were revealed. Furthermore, this study suggests the potentiality of coaching-and-training intervention toward perceived growth in IPC and self-compassion in ABA professionals. Initiating sustained conversation and competency training around emotionally responsive topics for ABA practitioners can support the social validity and acceptability of the ABA profession among individuals served including the autistic community. Continuing to engage current and future ABA practitioners around these topics can improve both workforce understanding and collaborative relationships with partners who serve alongside behavior-analytic practitioners.

## Data Availability

The datasets generated during and/or analyzed during the current study are not publicly available due to sensitivity and privacy of the data. Data may be available from the corresponding author on reasonable request, as deemed by the corresponding author.
